# Pitfalls and perils from FDA-approved germ-line cancer predisposition tests

**DOI:** 10.18632/oncotarget.28677

**Published:** 2024-12-24

**Authors:** Wafik S. El-Deiry, Eli Y. Adashi

**Keywords:** cancer predisposition, germline, marketing authorization, hereditary cancer, direct to consumer

## Abstract

The FDA approval on September 29, 2023, for “class III *de novo*” blood tests to assess hereditary cancer risk make widely available tests that may be obtained through a Direct to Consumer (DTC) path. There is concern that germ-line predisposition tests may not be reimbursed by insurance adding financial burdens to individuals and families. It is generally agreed in the fields of oncology and genetics that germ-line testing for disease susceptibility including cancer is best performed under care of a healthcare provider with genetic counseling. Our recommended cautions and call for change may seem paternalistic to some and may appear to infringe upon constitutional rights as they may relate to DTC, but there is a real concern with harm from germ-line testing of both adults and minors that can occur through DTC tests. The DTC option of germ-line testing for cancer susceptibility should be discouraged given the risks of anxiety, lack of adequate interpretation for variants not strongly associated with cancer, potential for minors to be tested outside the healthcare system and potential for loss of follow-up if test results are not shared with health care professionals or never make it into the medical record. The FDA should consider clear unambiguous guidance when it comes to germ-line DTC testing for cancer susceptibility for adults and especially for minors.

On September 29, 2023 the U.S. FDA granted “First Marketing Authorization” for a “class III *de novo*” test to assess risk of hereditary cancer [1]. Hereditary cancers include familial polyposis, Lynch Syndrome, breast and ovarian cancer, Li-Fraumeni Syndrome, gastric cancer, multiple endocrine neoplasia, von Hipple-Lindau Disease, mesothelioma, neurofibromatosis, tuberous sclerosis, and others [2]. The new FDA-approval for “Invitae Common Hereditary Cancers Panel” analyzes 48 genes and can be deployed as Direct-to-Consumer (DTC) test. The test uses blood, saliva, buccal-swab, or genomic DNA to detect mutations interpreted by Invitae’s internal gene variants database. Some alterations are classified as variants of uncertain significance (VUS) [3]. While the test poses minimal physical risk, there is no assurance of “safety” from psychological distress [4]. The test has not been shown as “effective” in improving patient outcomes or preventing cancers. While some strategies could prevent certain cancers with mismatch repair deficiency, genes such as *TP53* have no FDA-approved drugs or preventative vaccines. Invitae allows DTC testing online by inviting individuals with “option to (directly) initiate a test online through Invitae’s website.” The new approval route makes it easier for companies with similar panels to secure FDA-approval [1].

Genetic tests on common variants are not recommended in clinical practice and are not created equal regarding future cancer risk. Variants of genes such as *APC* can lead to attenuated polyposis. The penetrance and potency of a variant would be discussed during genetic counseling. The DTC path may lead to severe anxiety for some in absence of counseling. Companies offering germ-line tests include Myriad Genetics and Ambry Genetics. Myriad has premarket FDA-approval for *BRCA1*/*BRCA2* tests while Ambry has pointed out that “FDA approval alone is not a basis for coverage.” FDA approval raises issues regarding how the tests are regulated. Of note, Fanconi genes are not part of Invitae’s 48 gene panel even though they are cancer-predisposing and heritable. However, Invitae’s larger panels include Fanconi genes.

While the views expressed here and the recommended cautions may seem paternalistic to some, and may appear to infringe upon some constitutional rights as they may relate to DTC, there is a real concern with observed practices in the field when it comes to testing minors [5].

FDA approval for “class III *de novo*” blood tests to assess hereditary cancer risk make widely available tests that may not be reimbursed by insurance, and that would be best performed under care of a healthcare provider. The DTC option should be discouraged given risk of anxiety, lack of adequate interpretation for variants not strongly associated with cancer, potential for minors to be tested outside the healthcare system with potential for loss of follow-up if test results are not shared with health care professionals or never make it into the medical record. FDA should consider clear and unambiguous guidance when it comes to germ-line DTC testing for cancer susceptibility for adults and especially for minors, including the importance of follow-up and counseling.

## References

[1] FDA Grants First Marketing Authorization for a DNA Test to Assess Predisposition for Dozens of Cancer Types. 2023. https://www.fda.gov/news-events/press-announcements/fda-grants-first-marketing-authorization-dna-test-assess-predisposition-dozens-cancer-types.

[2] Brown GR , et al. JAAPA. 2020; 33:10–16. 10.1097/01.JAA.0000721648.46099.2c. 33234888

[3] Chen E , et al. JAMA Netw Open. 2023; 6:e2339571. 10.1001/jamanetworkopen.2023.39571. 37878314 PMC10600581

[4] Oliveri S , et al. Front Genet. 2018; 9:624. 10.3389/fgene.2018.00624. 30619456 PMC6295518

[5] Howard HC , et al. Eur J Hum Genet. 2011; 19:1122–26. 10.1038/ejhg.2011.94. 21629297 PMC3198149

